# Impact of Acute Energy Drink Consumption on Heart Rate Variability in Children and Adolescents. A Randomized Trial

**DOI:** 10.1007/s00246-025-03770-3

**Published:** 2025-01-21

**Authors:** Guido Mandilaras, Pengzhu Li, Robert Dalla-Pozza, André Jakob, Nikolaus Alexander Haas, Felix Sebastian Oberhoffer

**Affiliations:** 1 Department of Pediatric Cardiology and Pediatric Intensive Care, University Hospital of Munich, Ludwig Maximilians University München, 81377 Munich, Germany; 2 Center for Congenital Heart Defects Stuttgart, Pediatric Intensive Care, Pneumology and Allergology, Klinikum Stuttgart, Olgahospital/Women’s Clinic, Kriegsbergstrasse 62, 70174 Stuttgart, Germany

**Keywords:** Energy drinks, Electrocardiography, Heart rate variability, Children, Adolescents

## Abstract

The EDUCATE study investigated the acute impact of energy drink (ED) consumption on heart rate variability (HRV) in children and adolescents, with a focus on how these stimulant-rich beverages influence cardiac autonomic function. Given the popularity of EDs among young people, this study assessed the immediate cardiovascular response to ED intake. This randomized, double-blind, placebo-controlled crossover trial involved 26 healthy participants aged 10–18 years. Each participant consumed a weight-adjusted ED or placebo in two separate test sessions. HRV was monitored via a 24-h Holter ECG, with analysis centered on time-domain measures, such as the standard deviation of normal RR intervals (SDNN) and root-mean square of successive differences (RMSSD), along with frequency-domain metrics. Statistical analysis included a two-way repeated-measures ANOVA to examine the effects of “beverage” and “time.” The study revealed a significant increase in the SDNN in the ED group within the first hour post-consumption, with a mean difference of 17.692 ms compared with that in the placebo group (SDNN: ED = 133.346 ± 50.217 ms vs. placebo = 115.654 ± 47.583 ms, *p* = 0.023). However, no significant differences in the SDNN were observed in the subsequent time intervals (60–240 min). In addition, frequency-domain parameters, such as total power, RMSSD, LF, HF, and the LF/HF ratio, showed no significant changes across the four-hour observation period, indicating that sympathetic activation was transient. The findings suggest that ED consumption in children and adolescents leads to a temporary increase in autonomic activity, marked by elevated SDNN, without lasting dysregulation. While the cardiovascular effects are brief, acute sympathetic activation underscores the need for regulated ED intake among minors. Further studies are recommended to explore the long-term effects of regular ED consumption on cardiovascular health in youth.

## Introduction

Energy drinks (EDs) have become a popular beverage choice, particularly among children and adolescents, despite well-documented concerns over their potential adverse effects on the cardiovascular system [1]. These beverages contain high levels of caffeine and other stimulants, such as taurine and guarana, which are marketed to enhance mental and physical performance. However, numerous studies have demonstrated that excessive ED consumption can lead to adverse cardiovascular effects, including increased blood pressure, arrhythmias, and an altered heart rate [1–5].

The EDUCATE study, a comprehensive research initiative conducted at the University Hospital of Munich, aimed to examine the acute effects of ED consumption on pediatric cardiovascular function. Previous investigations by our working group highlighted the impact of ED consumption on blood pressure [3, 4], arterial stiffness [5], heart rhythm [2], and left ventricular efficiency [6].

HRV is recognized as a vital tool for assessing cardiovascular health. By analyzing heartbeat variations, HRV provides insights into autonomic nervous system function, particularly the balance between sympathetic and parasympathetic control, which are key indicators of cardiac well-being. For example, HRV can detect early cardiovascular abnormalities, making it an invaluable noninvasive measure in clinical practice [7]. Time-domain metrics, such as the SDNN, add predictive value for assessing cardiac risk [8]. In addition, the ability of the HRV to evaluate adaptability and recovery further enhances its utility across clinical, preventive, and athletic settings, making it indispensable for proactive cardiovascular care [9].

To the best of our knowledge, the impact of acute ED ingestion on pediatric HRV, a key marker of autonomic regulation, has not yet been addressed.

## Materials and Methods

### Ethical Statement

The study was conducted in accordance with the guidelines of the Declaration of Helsinki and was approved by the Ethics Committee of Ludwig Maximilians University Munich (Munich, Germany) (protocol code: 20–0993, date of approval: 12 January 2021). Prior written informed consent was obtained from all study participants, and in the case of minor participants, additional consent was obtained from parents or legal guardians.

### Study Population and Design

The EDUCATE study was designed as a randomized, double-blind, placebo-controlled crossover trial. A total of 27 healthy children and adolescents aged 10–18 years were recruited. The exclusion criteria included the presence of chronic conditions, a history of sudden heart death within the family, known allergies against beverage ingredients, regular use of medication with effects on cardiovascular function, regular use of drugs, including smoking and alcohol consumption, and pregnancy. Each participant consumed a weight-adjusted dose of an ED (3 mg caffeine per kg body weight) and a placebo beverage on two consecutive days. The caffeine dose represented the maximal daily dose of caffeine for minors as recommended by the European Food Safety Authority [10]. Both beverages were matched in volume. According to the product label, the ED contained caffeine (32 mg/100 mL), taurine (200 mg/100 mL), glucuronolactone (24 mg/10 mL), ginseng aroma extract (10 mg/100 mL), guarana extract (10 mg/100 mL), and vitamins. The placebo drink contained carbonated water, multifruit juice, and fruit and vegetable extracts. Both beverages had similar sugar contents (ED: 15.2 g/100 mL, placebo drink: 13.2 g/100 mL) and tastes. Both beverages were administered in identical and masked drinking bottles at room temperature on two consecutive days. Study participants were not allowed to consume any sources of caffeine (e.g., coffee, tea, chocolate) or drugs (e.g., tobacco, alcohol) 48 h before and 24 h after participation. Overnight fasting (apart from water) was requested before every study day. Finally, participants were expected not to consume any food or liquids during each examination period.

HRV was monitored via 24-h Holter electrocardiograms (ECGs) within a four-hour period. Data were recorded in the supine position via a portable 3-lead Holter ECG device (CardioMem® CM 4000, Hercules, Teltow, Germany). The electrodes were positioned according to conventional ECG documentation guidelines, as all included study participants displayed normal cardiac anatomy [11]. ECG data were evaluated offline by two blinded researchers.

### Heart Rate Variability Analysis

HRV analysis was conducted via standard time-domain and frequency-domain parameters, including the standard deviation of all normal RR intervals (SDNN), the root-mean square of successive differences (RMSSD), and low-frequency (LF) and high-frequency (HF) components. These parameters were evaluated within a four-hour period for the following time intervals: 0–60, 60–120, 120–180, and 180–240 min.

To minimize circadian rhythm changes, beverages were administered at similar morning hours on both days [12]. Moreover, the study participants were asked to remain in the supine position for the entire duration of the examination to minimize the influence of physical activity on the recorded cardiovascular parameters.

Our study employed a structured HRV analysis framework to comprehensively evaluate autonomic responses to ED consumption. This approach encompassed three core domains: (1) heart rate analysis, as a primary cardiovascular and HRV determinant; (2) global HRV indices, including SDNN and total power, to assess overall autonomic flexibility; and (3) vagal-specific indices such as RMSSD and HF power, reflecting parasympathetic regulation. By integrating these dimensions, we provided a holistic view of the autonomic shifts induced by EDs.

### Statistical Analysis

As this was a pediatric pilot study, pediatric values for ED-induced changes in the autonomic nervous system were not available and could not be included in the power analysis. Histograms, QQ plots, and the Shapiro‒Wilk test were used to test the normal distribution of continuous variables. The mean and standard deviation (SD) are used for all continuous variables. The ordinal and nominal variables are presented as percentages and counts. Sqrt or Ln data transformations were used when the data were not normally distributed. Two-way repeated measures’ analysis of variance (ANOVA) was applied to assess the main effects of “beverage,” “time,” and the interaction of “beverage and time” on the SDNN, total power, LF, HF, and LF/HF ratio. For post hoc testing, the Bonferroni adjusted pairwise test was used. The data were analyzed independently by a blinded statistician via SPSS (IBM SPSS Statistics for Windows, version 26.0. IBM Corp., Armonk, NY, USA). Statistical significance was defined as a *p* value < 0.05.

## Results

### Patient Characteristics

A total of 26 healthy children and teenagers were included in the analysis. The patients’ characteristics are shown in Table 1. None of the patients had medical conditions or were taking any medications. Twelve out of the twenty-six study participants (46.15%) correctly guessed the day of ED administration, suggesting appropriate blinding quality.

**Table 1:** Study participants’ characteristics (*n* = 26)

Characteristics	Total
Age (years), mean (SD)	14.49 ± 2.44
Sex, *n* (%)	
Male	13 (50)
Female	13 (500)
Weight Classification, *n* (%)	
Normal weight	22 (84.62)
Overweight	4 (15.38)
Obese	0 (0)
Caffeine consumption Behavior, *n* (%)^a^	
Rarely	16 (61.54)
Occasionally	3 (11.54)
Frequently	5 (19.23)
Daily	2 (7.69)
Energy Drink consumption Behavior, *n* (%)^b^	
Never	11 (42.31)
Rarely	11 (42.31)
Occasionally	1 (3.84)
Frequently	3 (11.54)
Daily	0 (0)

^a^Rare caffeine consumers if < 1 caffeine containing drink per month, occasional caffeine consumers if 1–3 drinks per month, frequent caffeine consumers if 1–6 caffeine containing drinks per week and daily caffeine consumers if ≥ 1 caffeine containing drink per day (https://doi.org/10.1161/JAHA.118.011318)

^b^Energy Drink (ED) consumption behavior was evaluated as follows: Rare ED consumers if < 1 ED per month, occasional ED consumers if 1–3 EDs per month, frequent ED consumers if 1–6 EDs per week and daily ED if ≥ 1 ED per day

### Mean HR

The interaction between beverage and time had a statistically significant effect on the mean HF (*p* < 0.001). Therefore, the separate effect of beverage variable was analyzed at each time interval.

The mean HR was demonstrated to be lower in the ED group compared to the placebo group two hours after the beverage consumption with a difference of 2.71bpm, *p* = 0.012.

**Fig. 1:**
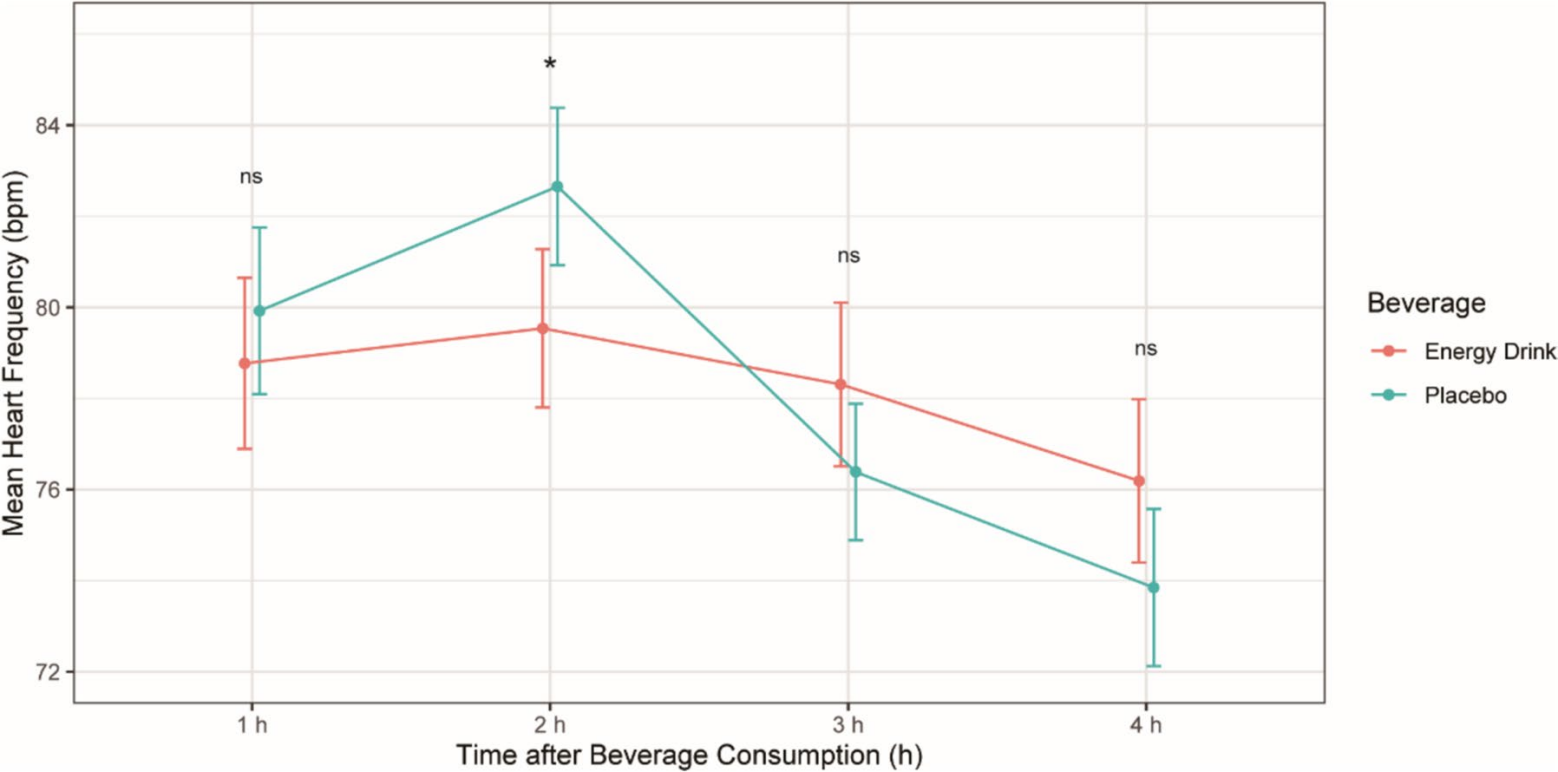
Mean heart rate (bpm) after energy drink and placebo consumption at different time points. *p < *0.05*

One hour, three hours, and four hours after beverage consumption, the differences in the mean HR between the subjects of the ED group and the placebo group were not statistically significant (*p* > 0.05). [Table 2, Fig. 1].

**Table 2:** The separate effect of beverage on mean HF (*n* = 26)

Parameters	Energy drink (bpm)	Placebo (bpm)	*p* value
Time 1 h	78.77 ± 9.58	79.92 ± 9.32	0.333
Time 2 h	79.54 ± 8.85	82.65 ± 8.81	0.012*
Time 3 h	78.31 ± 9.18	76.39 ± 7.64	0.125
Time 4 h	76.19 ± 9.14	73.85 ± 8.79	0.095

Mean ± standard deviation is used for normally distributed variables. * *p* < 0.05

### Heart Rate Variability Analysis

#### SDNN

**Fig. 2:**
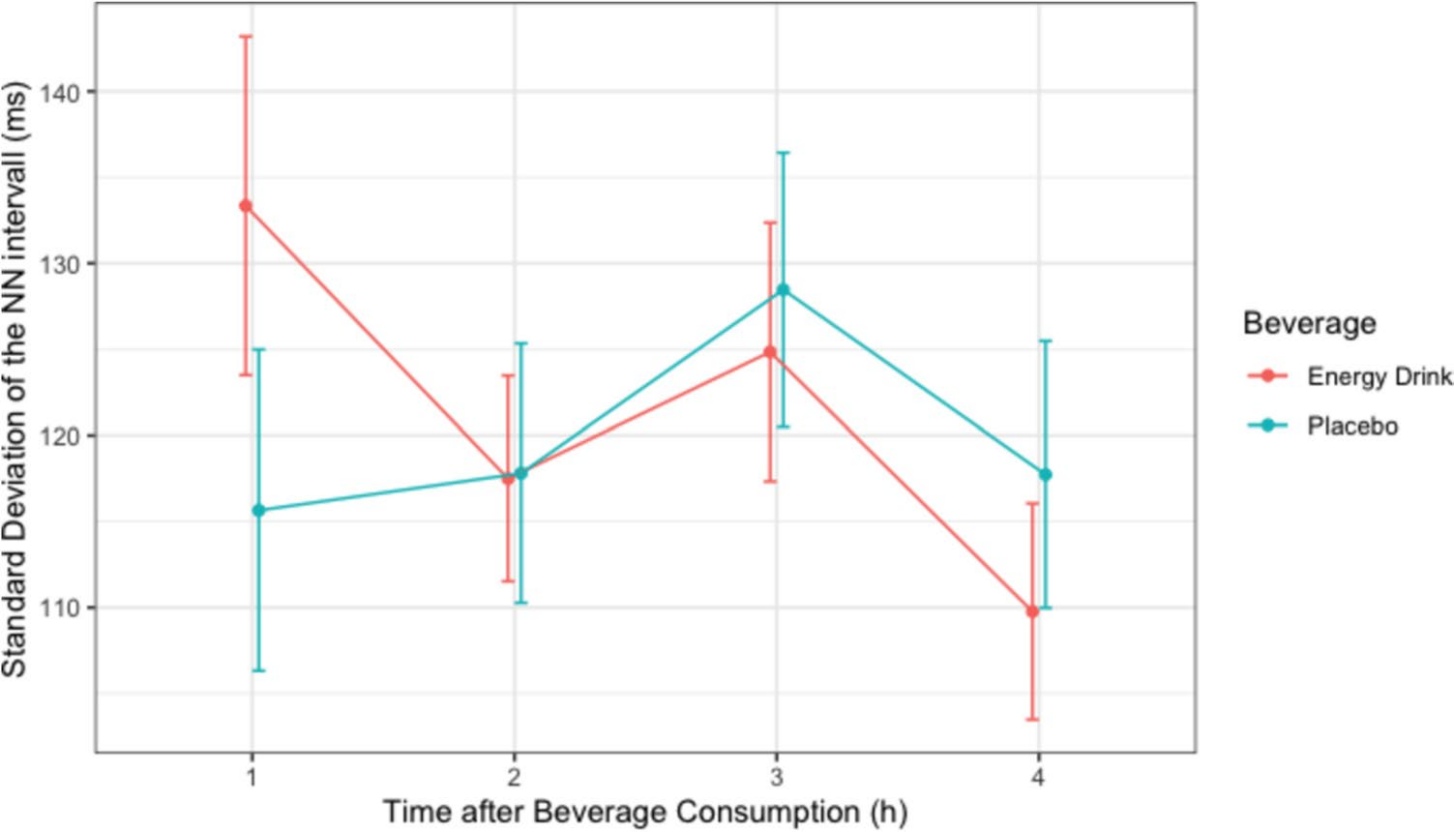
The separate effect of beverage on SDNN (*n* = 26)

The Shapiro–Wilk test revealed a non-normal distribution of SDNN in the ED group for the time period 0–60 min after consumption. Non-normally distributed data were transformed into normally distributed Sqrt-form for further analysis. After Mauchly’s spherical hypothesis test for the interaction term “beverage and time,” the variance and covariance matrices of the dependent variables were equal (*p* > 0.05). The interaction between “beverage and time” had a statistically significant effect on the SDNN (*p* = 0.015). Therefore, the separate effects of the variable “beverage” were analyzed at each time interval. The SDNN was greater in the ED group than in the placebo group from 0 to 60 min after beverage consumption, with a mean difference of 17.692 ms (*p* = 0.023) [Table 3, Fig. 2]. The remaining time periods did not significantly differ in the SDNN between the two beverages.

**Table 3:** The separate effect of beverage on SDNN (*n* = 26)

Parameters	Energy drink (ms)	Placebo (ms)	*p* value
Time 1h	133.346 ± 50.217	115.654 ± 47.583	0.023*
Time 2h	117.500 ± 30.448	117.808 ± 38.426	0.950
Time 3h	124.846 ± 38.324	128.462 ± 40.636	0.407
Time 4h	109.769 ± 32.057	117.731 ± 39.534	0.081

Mean ± standard deviation is used for normally distributed variables. * *p* < 0.05

Our findings indicated that SDNN, a time-domain measure of HRV, was significantly affected following ED consumption. During the first hour, the SDNN increased significantly compared with that of the placebo (*p* < 0.05), indicating a heightened HRV and reflecting an acute response of the autonomic nervous system (ANS) to energy drink consumption [13]. This acute increase is likely due to the stimulating effects of caffeine and other active ingredients in energy drinks.

### Total Power

**Fig. 3:**
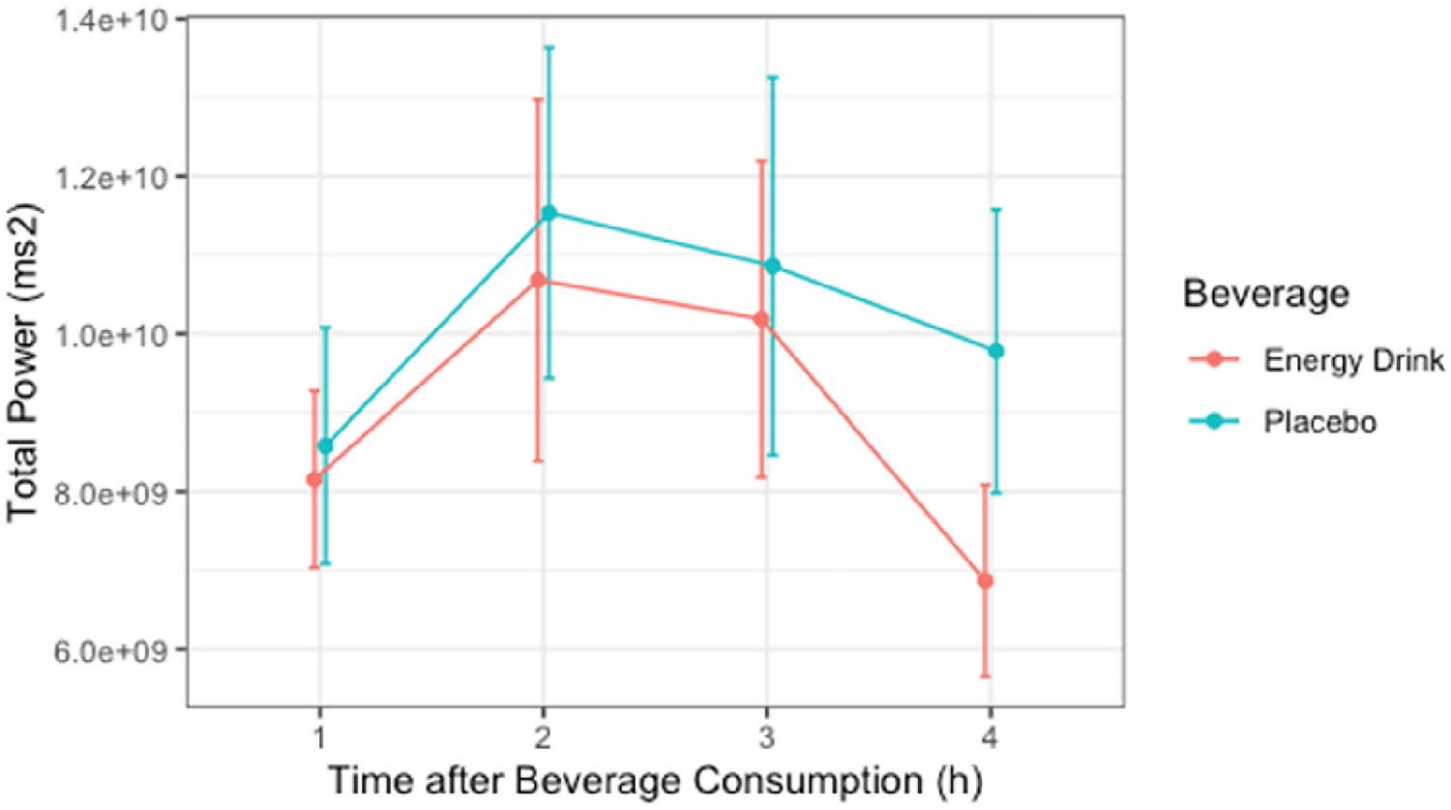
Total power/1000000 (*n* = 26)

The Shapiro–Wilk test revealed non-normal distribution of total power in both groups for all the time periods. Non-normally distributed data were transformed into normally distributed Lg-form for further analysis. The interaction between “beverage and time” had no statistically significant effect on total power (*p* > 0.05). In addition, the main effect of the variable “beverage” on total power did not show significant difference (*p* > 0.05) [Table 4, Fig. 3].

**Table 4:** Total power/1000000 (*n* = 26)

Parameters	Energy drink (ms^2^)	Placebo (ms^2^)
Time 1h	8155 ± 5715	8581 ± 7633
Time 2h	10,681 ± 11,693	11,536 ± 10,721
Time 3h	10,187 ± 10,229	10,861 ± 12,231
Time 4h	6868 ± 6211	9779 ± 9174

Data are presented as mean ± SD

### LF

**Fig. 4:**
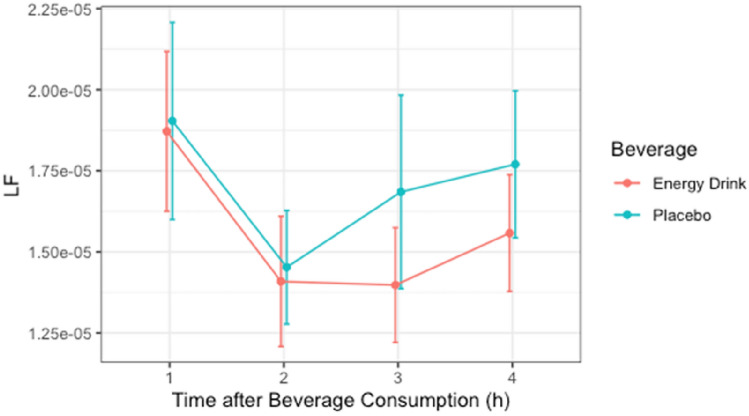
LF*10,000 (*n* = 26)

The Shapiro–Wilk test revealed non-normal distribution of LF for the time periods 0–60, 60–120, 120–180 min after consumption in the ED group and for the time periods 0–60, 120–180, and 180–240 min after consumption in the placebo group. Non-normally distributed data were transformed into normally distributed Sqrt-form for further analysis. After Mauchly’s spherical hypothesis test for the interaction term “beverage and time,” the variance and the covariance matrices of the dependent variables were equal (*p* > 0.05). The interaction between “beverage and time” had no statistically significant effect on LF (*p* > 0.05). In addition, the main effect of the variable “beverage” on total power did not show significant difference (*p* > 0.05) [Table 5, Fig. 4].

**Table 5:** LF*10,000 (*n* = 26)

Parameters	Energy drink	Placebo
Time 1h	0.19 ± 0.13	0.19 ± 0.16
Time 2h	0.14 ± 0.10	0.15 ± 0.09
Time 3h	0.14 ± 0.09	0.17 ± 0.15
Time 4h	0.16 ± 0.09	0.18 ± 0.12

Data are presented as mean ± SD

### HF

**Fig. 5:**
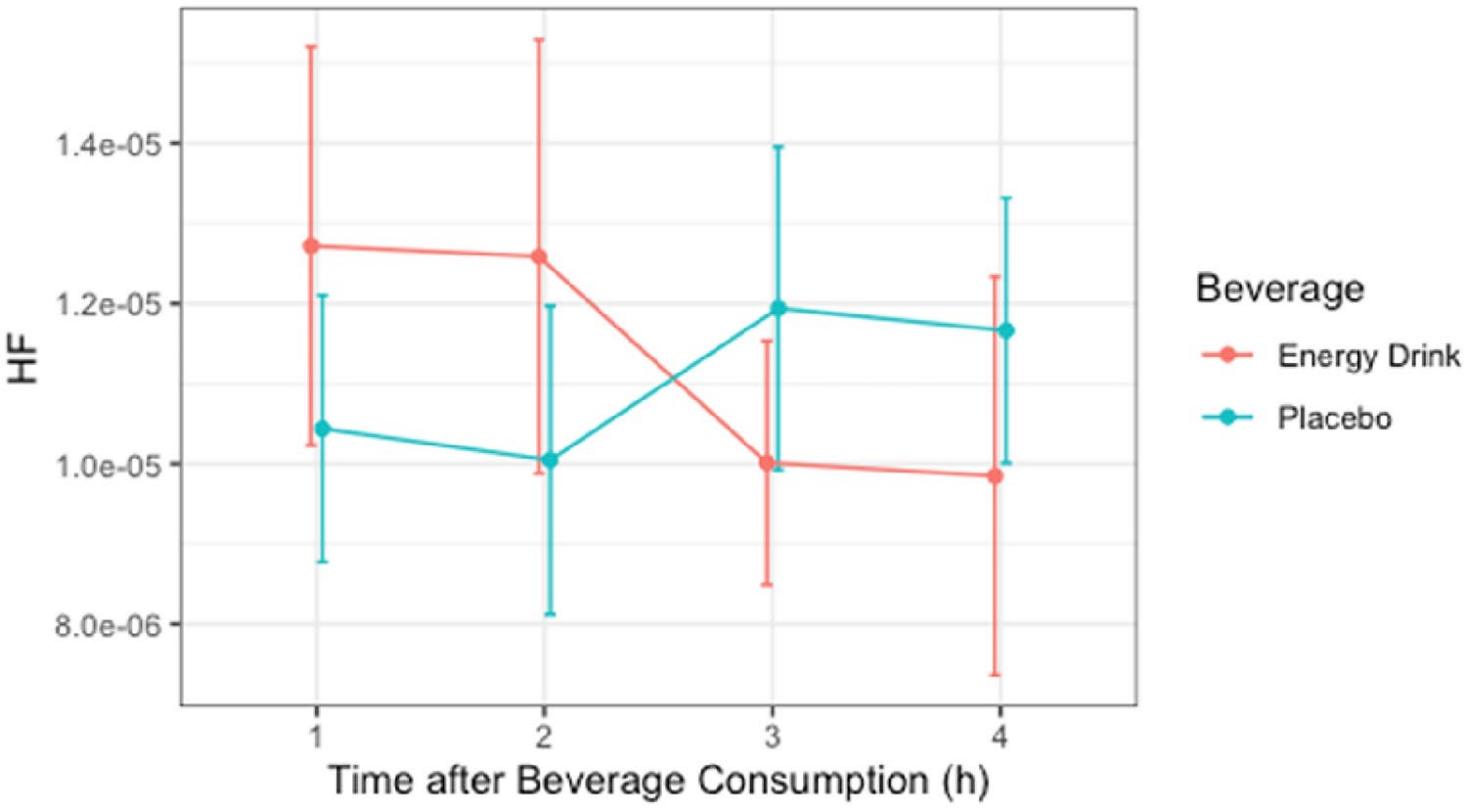
HF*10,000 (*n* = 26)

The Shapiro–Wilk test revealed non-normal distribution of power in both groups for all the time periods. Non-normally distributed data were transformed into normally distributed Lg-form for further analysis. After Mauchly’s spherical hypothesis test for the interaction term “beverage and time,” the variance and the covariance matrices of the dependent variables were equal (*p* > 0.05). The interaction between “beverage and time” had no statistically significant effect on HF (*p* > 0.05). In addition, the main effect of the variable “beverage” on HF did not show significant difference (*p* > 0.05) [Table 6, Fig. 5].

**Table 6:** HF*10,000 (*n* = 26)

Parameters	Energy drink	Placebo
Time 1h	0.127 ± 0.127	0.104 ± 0.085
Time 2h	0.126 ± 0.138	0.100 ± 0.098
Time 3h	0.100 ± 0.078	0.119 ± 0.102
Time 4h	0.099 ± 0.127	0.117 ± 0.084

Data are presented as mean ± SD

### LF/HF Ratio

**Fig. 6:**
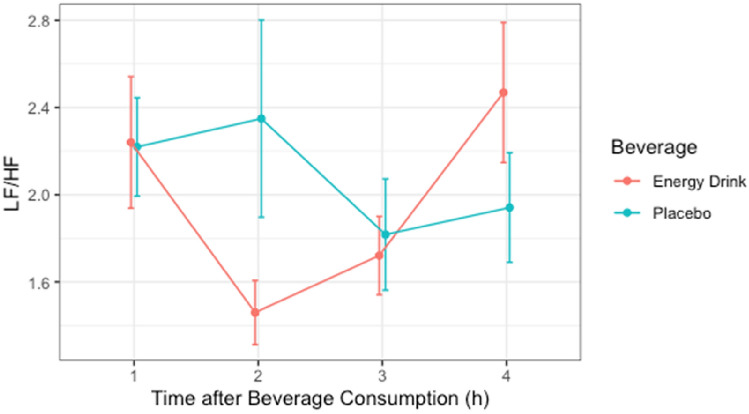
LF/HF (*n* = 26)

The Shapiro–Wilk test revealed non-normal distribution of power for the time period 0–60, 180–240 min in the ED group and time period 60–120, 120–180, and 180–240 min in the placebo group. Non-normally distributed data were transformed into normally distributed Sqrt-form for further analysis. After Mauchly’s spherical hypothesis test for the interaction term “beverage and time,” the variance and the covariance matrices of the dependent variables were equal (*p* > 0.05). The interaction between “beverage and time” had no statistically significant effect on LF/HF (*p* > 0.05). In addition, the main effect of the variable “beverage” on total power did not show significant difference (*p* > 0.05) [Table 7, Fig. 6].

**Table 7:** LF/HF (*n* = 26)

Parameters	Energy drink	Placebo
Time 1h	2.24 ± 1.54	2.22 ± 1.15
Time 2h	1.46 ± 0.75	2.35 ± 2.31
Time 3h	1.72 ± 0.92	1.82 ± 1.30
Time 4h	2.47 ± 1.64	1.94 ± 1.28

Data are presented as mean ± SD

### RMSSD

The Shapiro–Wilk test revealed normal distribution of RMSSD in both groups. Paired-t test shows no statistically significant differences between two groups [Table 8].

**Table 8:** Effect of beverage consumption on RMSSD over 240 min (*n* = 26)

Parameters	Energy drink (ms)	Placebo (ms)	*p* value
RMSSD (ms)	72.38 ± 31.28	70.10 ± 25.24	0.723

Mean ± standard deviation is used for normally distributed variables. Median (IQR) is used for non-normally distributed variables

No significant differences were observed in total power, LF, HF, or the LF/HF ratio and RMSSD across the four-hour post-consumption period. This suggests that while ED consumption may induce short-term changes in certain HRV parameters (such as SDNN), it does not cause prolonged alterations in autonomic balance, as reflected by frequency-domain measures.

## Discussion

The present study delves into the acute effects of ED consumption on HRV in children and adolescents. These findings suggest that EDs have a notable but transient impact on certain HRV parameters, specifically heart rate and SDNN, during the initial period post-consumption. These results align with other studies investigating the cardiovascular effects of EDs in both pediatric and adult populations.

Pstras (2023) reported that ED leads to reduced HRV in young adults after exercise, suggesting an influence on autonomic balance, potentially increasing sympathetic over parasympathetic activity, which supports our findings of autonomic shifts post-consumption [14]. Similarly, Nw et al. (2020) reported that both men and women presented reduced HRV and altered recovery patterns postexercise with ED intake, implying delayed autonomic recovery [15]. Both studies examined the effects of ED consumption on HRV following exercise, highlighting a trend toward decreased HRV post-consumption. Together, these studies indicate that ED may hinder cardiac autonomic regulation during postexercise recovery, emphasizing the need for caution in their use.

### HRV in Children and Adolescents with Health Conditions

The impact of disease on heart rate variability (HRV) in children and adolescents is a crucial area of research, revealing insights into autonomic nervous system function and potential cardiovascular risks. Studies have shown that HRV is significantly influenced by various conditions, indicating autonomic dysregulation in pediatric populations. For example, Zhou et al. (2012) reported a strong correlation between cardiovascular risk factors and autonomic nervous system activity in children, suggesting that increased risk factors are associated with decreased HRV. This reduction may signify an imbalance between sympathetic and parasympathetic responses, making HRV a useful marker for cardiovascular health in young patients [16].

Research by Azak and Cetin (2021) specifically highlights autonomic dysregulation in children with structurally normal hearts but with premature cardiac beats, implying that HRV can detect early dysregulation even without structural anomalies [17].

A significant HRV measure, the standard deviation of normal-to-normal intervals (SDNN), provides value in assessing overall autonomic function and health risks in these populations. For example, Connell et al. (2024) reported that children with acute decompensated heart failure displayed decreased HRV, particularly at lower SDNN values, which correlated with poorer outcomes [18]. This reduction in the SDNN indicates diminished autonomic flexibility, which can be predictive of clinical severity and recovery challenges. Leppänen et al. (2020), in the PANIC study, further supported the association between low SDNN and cardiometabolic risk factors, underscoring the role of SDNN in detecting early signs of autonomic dysfunction [19].

Finally, Latus et al. (2015) explored HRV in pediatric pulmonary hypertension patients and reported that a reduced HRV, especially in the SDNN, corresponded with increased disease severity [20]. Taken together, these studies emphasize HRV, particularly SDNN, as a noninvasive, sensitive indicator of disease impact on the autonomic nervous system in children, making it invaluable for early diagnosis and monitoring of disease progression in pediatric care.

### Heart Rate and Supraventricular Extrasystoles (SVES) After ED Consumption

ED intake resulted in a significant increase in SVES compared to a placebo, highlighting its arrhythmogenic potential. However, mean heart rate was lower in the ED group during 60–120 min post-consumption [2]. This paradoxical response may reflect reflexive autonomic adjustments to increased blood pressure induced by EDs [3, 4]. No significant changes in QTc intervals were observed, suggesting limited immediate risks under controlled dosages.

### SDNN and Beverage Consumption

Our results demonstrated a significant increase in SDNN during the first hour following ED consumption compared with placebo. This acute increase in the SDNN is likely due to the stimulating effects of caffeine, which has been shown to activate the sympathetic nervous system, leading to changes in heart rate, myocardial contractility, and peripheral vasoconstriction [2–6, 21]. This finding indicates an immediate autonomic response to ED intake, driven largely by caffeine’s well-known effects on the cardiovascular system. Higgins et al. (2010) emphasized that caffeine and taurine, the primary components of energy drinks, are potent cardiovascular stimulants, with acute effects on blood pressure and HRV that peak shortly after consumption [22].

In contrast, studies conducted on adults have demonstrated a more variable response to ED consumption, with some reporting increases in HRV and others reporting decreases, depending on the study design and population characteristics. For example, Grasser et al. (2014) reported that young adults presented elevated blood pressure, a double product and a lower cerebral velocity following ED consumption, indicating reduced parasympathetic activity and heightened sympathetic tone [23]. Nelson et al. (2014) conducted a double-blind, placebo-controlled study on the effects of a modified ED and reported that while it significantly increased the resting heart rate, it had no significant effect on heart rate variability or exercise capacity in the subjects [24]. The discrepancy between adolescents and adults could be attributed to differences in baseline autonomic tone, caffeine metabolism, and cardiovascular reactivity between age groups.

### Absence of Long-Term Effects

While the SDNN was significantly elevated in the first hour post-consumption, no significant changes were observed in subsequent time intervals (60–240 min). This suggests that the effect of EDs on HRV is transient, with a return to baseline levels within a few hours. According to Seifert et al. (2011), the cardiovascular effects of EDs in children and adolescents typically dissipate within 2–4 h, aligning with the half-life of caffeine in the body [21]. Porto et al. (2021) similarly reported that ED consumption did not impact autonomic recovery after moderate aerobic exercise, reinforcing the notion that the effects of EDs are short-lived in certain contexts [25].

However, studies in adults have occasionally shown more prolonged effects. For example, Steinke et al. (2009) reported that in healthy adults, ED consumption led to sustained increases in blood pressure and alterations in HRV for up to six hours [26].

### Total Power, RMSSD, LF, HF, and the LF/HF Ratio

In our study, no significant differences were observed in total power, RMSSD, LF, HF, or the LF/HF ratio during the four-hour post-consumption observation period. This finding is consistent with studies on young adults that reported no significant changes in these frequency-domain parameters. For example, Steinke et al. (2009) reported that while heart rate increased after ED consumption, there were no consistent changes in LF or HF components [26].

The analysis of vagal-related parameters, particularly RMSSD and HF power, offers critical insights into parasympathetic influences following energy drink (ED) consumption. RMSSD, a time-domain measure, reflects short-term variations in heart rate driven by vagal modulation. In our study, RMSSD values did not differ significantly between ED and placebo groups, indicating limited parasympathetic withdrawal post-consumption. Similarly, HF power, a frequency-domain parameter closely tied to respiratory-related vagal activity, remained stable across all time intervals. These findings suggest that while EDs elicit transient sympathetic activation, parasympathetic tone is relatively preserved in the immediate aftermath. These results align with prior studies highlighting that caffeine-based stimulants predominantly affect sympathetic pathways without significantly impairing vagal function [22, 26].

### Implications for Adolescent Cardiovascular Health

The observed transient increase in SDNN highlights heightened autonomic responsiveness, while stable RMSSD and HF power suggest preserved vagal tone, underscoring the nuanced interplay between sympathetic and parasympathetic dynamics in acute scenarios [13, 25].

The acute increase in SDNN observed in this study suggests that the cardiovascular system in adolescents is capable of responding rapidly to the stimulant effects of EDs. However, the safety of repeated or long-term ED consumption in this population remains a concern. Adolescents are at a developmental stage where their autonomic and cardiovascular systems may be more sensitive to external stimuli, raising concerns about potential adverse effects.

Given that no significant changes in RMSSD and the LF/HF ratio were observed, we can infer that ED consumption in healthy adolescents may not lead to prolonged autonomic imbalance. Nevertheless, as Seifert et al. (2011) suggested, those with underlying cardiovascular conditions may be at greater risk for adverse outcomes following ED consumption [21].

### Public Health Implications and Future Directions

The widespread availability of energy drinks among adolescents raises important public health concerns. Although the effects observed in this study were transient, they underscore the need for clearer guidelines regulating ED consumption in minors. Excessive intake of EDs can lead to arrhythmias, elevated blood pressure, and autonomic dysfunction in susceptible individuals [26].

We previously demonstrated that ED consumption is associated with lower left ventricular efficiency and elevated blood pressure in children and adolescents, highlighting the importance of regulating access to these beverages among minors [2–7]. In addition, Svatikova et al. (2015), in a randomized, double-blind, placebo-controlled study, reported that EDs significantly increased systolic and diastolic blood pressure and norepinephrine levels but had no effect on heart rate or stress response and that those beverages could pose significant health risks to younger populations, especially those with undiagnosed cardiovascular issues, owing to their potent-stimulant effects [27].

## Conclusion

The EDUCATE study demonstrated that acute consumption of EDs significantly altered the cardiac rhythm, specifically by decreasing the heart rate, increasing the number of SVES [2] and increasing the SDNN. The acute rise in SDNN suggests a heightened autonomic response, likely driven by caffeine and other stimulants in EDs, which transiently increase sympathetic activity. While these effects were pronounced in the first hours after consumption, they dissipated within four hours, indicating a temporary impact on the HRV.

This transient autonomic activation may not pose a significant risk to healthy adolescents. Studies in both pediatric and adult populations, such as those by Higgins et al. (2010) and Seifert et al. (2011), have consistently highlighted the cardiovascular risks associated with excessive or regular ED consumption, particularly among vulnerable individuals [21, 22]. Long-term or repeated exposure to these stimulants may lead to adverse cardiovascular events, making it essential to establish regulatory guidelines for ED consumption, particularly in minors.

Further research is needed to investigate the chronic effects of habitual ED consumption on cardiovascular health in this age group. In addition, public health interventions are necessary to limit the availability and consumption of EDs, particularly among young individuals, who may be more susceptible to adverse effects.

## Data Availability

No datasets were generated or analysed during the current study.
